# The Joint Effect of Sleep Duration and Disturbed Sleep on Cause-Specific Mortality: Results from the Whitehall II Cohort Study

**DOI:** 10.1371/journal.pone.0091965

**Published:** 2014-04-03

**Authors:** Naja Hulvej Rod, Meena Kumari, Theis Lange, Mika Kivimäki, Martin Shipley, Jane Ferrie

**Affiliations:** 1 Department of Public Health, University of Copenhagen, Copenhagen, Denmark; 2 Copenhagen Stress Research Center, Copenhagen, Denmark; 3 Department of Epidemiology and Public Health, University College London, London, England; 4 School of Social and Community Medicine, University of Bristol, Bristol, England; Kagoshima University Graduate School of Medical and Dental Sciences, Japan

## Abstract

**Background:**

Both sleep duration and sleep quality are related to future health, but their combined effects on mortality are unsettled. We aimed to examine the individual and joint effects of sleep duration and sleep disturbances on cause-specific mortality in a large prospective cohort study.

**Methods:**

We included 9,098 men and women free of pre-existing disease from the Whitehall II study, UK. Sleep measures were self-reported at baseline (1985–1988). Participants were followed until 2010 in a nationwide death register for total and cause-specific (cardiovascular disease, cancer and other) mortality.

**Results:**

There were 804 deaths over a mean 22 year follow-up period. In men, short sleep (≤6 hrs/night) and disturbed sleep were not independently associated with CVD mortality, but there was an indication of higher risk among men who experienced both (HR = 1.57; 95% CI: 0.96–2.58). In women, short sleep and disturbed sleep were independently associated with CVD mortality, and women with both short and disturbed sleep experienced a much higher risk of CVD mortality (3.19; 1.52–6.72) compared to those who slept 7–8 hours with no sleep disturbances; equivalent to approximately 90 additional deaths per 100,000 person years. Sleep was not associated with death due to cancer or other causes.

**Conclusion:**

Both short sleep and disturbed sleep are independent risk factors for CVD mortality in women and future studies on sleep may benefit from assessing disturbed sleep in addition to sleep duration in order to capture health-relevant features of inadequate sleep.

## Introduction

Inadequate sleep is under-recognized as a problem in modern society, although it has been estimated that about one in five adults are affected by sleep problems [1]. Findings from two recent independent systematic reviews and meta-analyses show a U-shaped association between sleep duration and mortality with both short and long sleep being predictors of all-cause mortality [2], [3]. Associations between sleep duration and cause-specific mortality present a more mixed picture with associations between CVD, cancer and ‘other’ deaths observed for both short and long sleep in some, but not all, studies [4].

While most studies have focused on the health effects of sleep duration, poor sleep quality may also be important for health. Disturbed sleep is likely to suppress slow wave sleep, which facilitates general biological restoration, and a recent experimental study found that reduced sleep quality, without changes in sleep duration, was associated with decreased insulin sensitivity and glucose tolerance [5]. Only a few population studies have addressed the association between sleep disturbances and mortality. While there have been inconsistencies in the results [4], we have recently observed a relatively strong association between sleep disturbances and premature death in a cohort of French men and women [6].

While even less work has examined both sleep duration and sleep disturbances, findings from the Whitehall II study showed that the effect of short sleep duration on coronary heart disease (CHD) incidence was greatest among those with disturbed sleep [7]. This observation was supported by findings from a Dutch population-based study, which showed a higher risk of CHD incidence in short sleepers, especially if they also reported poor sleep quality [8]. Although these results suggest that sleep duration and sleep quality have synergistic effects on cardiovascular health, the findings for mortality are less clear. In a smaller study including 3,430 middle-aged Chinese adults, a higher risk of all-cause mortality was associated with frequent insomnia among those sleeping more than 9 hours per night, but not among those with shorter sleep duration [9]. In contrast, a study from the US including 1,741 men and women showed a markedly higher risk of all-cause mortality among men, but not women, with short sleep duration and insomnia; a finding that was mainly confined to men with existing hypertension or diabetes [10]. A different distribution of causes of deaths in the two studies may explain some of the heterogeneity in the results. In the present study we examined for the first time both the individual and joint effects of sleep duration and disturbed sleep on total and cause-specific (CVD, cancer and other) mortality in a large population-based study including more than 9,000 men and women.

## Material and Methods

### Study population

The target population of the Whitehall II study was all London-based office staff aged 35 to 55 years working in 20 civil service departments in 1985. With a response rate of 73%, the final cohort consisted of 10,308 participants. Baseline screening (Phase 1), between late 1985 and early 1988, involved a clinical examination and a self-administered questionnaire. Subsequent phases of data collection have alternated between postal questionnaire alone (all even-numbered phases), and postal questionnaire accompanied by clinical examination (all odd-numbered phases). Further details have been described elsewhere [11], 12. Data from all 10,308 participants at Phase 1 were used to exclude everyone with pre-existing chronic disease; cardiovascular disease, diabetes, cancer, or respiratory disorders (n = 1,077), and everyone who reported taking sleeping pills on a daily basis (n = 17). Participants with missing information on sleep (n = 73), other covariates (n = 33) or of uncertain vital status at the end of follow-up (n = 10) were also excluded, leaving 6,114 men and 2,984 women for the statistical analyses.

### Sleep duration and disturbed sleep

Sleep duration was measured at Phase 1 using a single question “*How many hours of sleep do you have on an average week night?*” Response categories were 5 hours or less, 6, 7, 8, and 9 hours or more. Disturbed sleep was assessed by one question from the General Health Questionnaire-30^16^ *“Have you recently been having restless, disturbed nights?”* The response categories were Not at all, No more than usual, Rather more than usual, and Much more than usual. For the analyses, the last two categories were combined due to small numbers in the ‘Much more than usual’ category. In order to assess the joint effect of sleep duration and disturbances, we constructed a new composite variable with the following categories: 7–8 hours and no sleep disturbances; 7–8 hours and disturbed sleep; ≤6 hours and no sleep disturbances; and ≤6 hours and disturbed sleep. Participants with 9 or more hours of sleep were not included in this composite variable due to their increased mortality risk compared to those sleeping 7–8 hours.

### Covariates

All covariates were measured at phase 1. *Sociodemographic factors* included age, marital status (married/cohabiting: yes/no), ethnicity (White/non-White), and civil service employment grade, which was categorized as low (clerical and administrative support staff), intermediate (professional and executive staff), or high (senior administrative staff and managers). *Health-related behaviors* included smoking (never, ex-, current smoker), high alcohol consumption (defined as ≥15 units/week for women and ≥22 units/week for men), and physical activity (0, 1–2, 3+ hours of moderate to vigorous physical activity per week). Physical inactivity was defined as 0 hours of moderate to vigorous activity per week. *Cardiovascular risk factors* were measured at the screening examination and included body mass index (BMI) as kg/m^2^, systolic blood pressure (mmHg), and total cholesterol (mmol/L). *Depression/anxiety* included self-reported nervous trouble or persistent depression in the preceding 12 months. As the measure on depression/anxiety was included after the beginning of the study these data were only available for a subset of the cohort.

### Follow-up

The participants were followed from the date of the Phase 1 examination until date of death (n = 804) or end of follow-up on 31^st^ of January 2010. Mortality follow-up was available through the National Health Services Central Registry. Registration of death within 5 days is a legal requirement in the UK and participants not registered were assumed to be alive. Death certificates were coded using the 9^th^ and 10^th^ revisions of the International Classification of Disease (ICD) and categorized as cardiovascular disease (ICD-9 codes 390–459 and ICD-10 codes I00-I99), cancer (ICD-9 codes 140–208 and ICD-10 codes C00-C97), and other deaths (all remaining codes).

### Ethical approval

Ethical approval for the Whitehall II study was obtained from the University College London Medical School committee on the ethics of human research.

### Statistical analysis

Cox proportional hazards models with age as the underlying time variable were used to analyze the data. All variables met the proportional hazards assumption. Initially, we estimated hazard ratios (HR) and 95% confidence intervals (CI) for all-cause mortality and cause-specific mortality according to sleep duration, disturbed sleep, and the joint effect of the two. To assess the absolute effects of these variables on mortality, we also used the additive hazards model, which is a flexible semi-parametric model for survival outcomes [13]. In that model, the hazard is modeled as a linear function of the explanatory variables and the effect estimates can therefore be directly interpreted as the number of additional cases associated with the explanatory variables. We tested the underlying assumption of age-invariant effects and found no indication of violation. All analyses were conducted separately for men and women.

In statistical terms, with λ*_i_(t)* denoting the hazard of the event in question (e.g. total mortality) for person *i* at age *t* the following models were employed
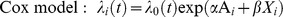


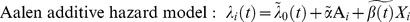
Where A*_i_* is the exposure status for person *i*; so in the analysis of sleep duration it would be a 5 level categorical variable and in the analysis of disturbed sleep it would be a 3 level categorical variable. *X_i_* denotes potential confounders (only included in the multiple adjusted analysis). The log hazard ratio is captured by α while the additive effect is captured by 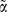. Finally, 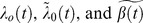 are unspecified age dependent functions. Using age as the time variable in both models allowed for thorough adjustment for confounding by age.

There is an ongoing discussion on how to define and measure interaction, but most health researchers would agree that interventions or preventive strategies should be aimed at patients or population sub-groups where most cases could potentially be prevented [14]. In order to identify such sub-groups, deviation from additivity of absolute effects is the relevant measure of interest. When applying additive hazards models, the explanatory models are fitted on an additive scale and product terms to assess deviation from additivity of effects can be included and used to directly obtain the number of additional deaths due to interaction [15].

A multiple adjusted model was fitted to adjust for confounding by baseline covariates. Potential confounders were identified based on existing knowledge on risk factors for impaired sleep and premature death using the methods of directed acyclic graphs [16] and included age, ethnicity, marital status, and employment grade. Menopausal status was also identified as a potential confounder among women, but as several women lacked information on menopausal status and it was not associated with total or CVD mortality in the current study, we decided not to include the variable in further analyses. As depression/anxiety was only assessed in a subset, we addressed potential confounding within this subset. Health-related behavior (alcohol, smoking, and physical activity), and cardiovascular risk factors (BMI, systolic blood pressure, and total cholesterol) are potential mediators of the relation between sleep and mortality, and adjusting for these variables could constitute over-adjustment (i.e. accounting for a variable on the causal pathway). We therefore only adjusted for health-related behavior and CVD risk factors in sensitivity analyses to see how such adjustments affected our results. In another sensitivity analysis, we also excluded the first four years of follow-up to prevent reverse causation by ill-health induced sleep problems. As we do not have information on sleep apnea, analyses in a sample restricted to those of normal weight (BMI<25 kg/m^2^), where the prevalence of sleep apnea is expected to be lower, were used to assess potential confounding by sleep apnea.

Additive hazards models were fitted using the software package R, version 2.13.1 (through the package “timereg”, version 1.5) and all other analyses were conducted using STATA, version 12.

## Results

### Baseline characteristics

Age at baseline ranged from 35 to 55 years with a mean of 45 years. Forty-four percent of the population reported either short sleep or disturbed sleep, with 9 percent reporting both (8% men and 11% women). Baseline characteristics of the population are shown in Table 1.

**Table 1:** Baseline Characteristics of 9,098 men and women free of pre-existing disease (cardiovascular disease, diabetes, cancer, respiratory disorders) from Phase 1 of the Whitehall II study.

	Total population	Men (N = 6114)				Women (N = 2984)			
		7–8 h sleep and no sleep disturbances (N = 3457)	Disturbed sleep only (N = 691)	≤6 h sleep only (N = 1456)	≤6 h sleep & disturbed sleep (N = 462)	7–8 h sleep and no sleep disturbances (N = 1553)	Disturbed sleep only (N = 406)	≤6 h sleep only (N = 664)	≤6 h sleep & disturbed sleep (N = 323)
Deaths, n	804	291	46	150	46	124	32	76	29
Baseline age, years	45	44	44	45	44	45	45	47	47
Lowest employment grade, n (%)	1976 (22)	264 (8)	41 (6)	183 (13)	42 (9)	766 (49)	161 (40)	327 (49)	156 (48)
Married, n (%)	6776 (74)	2825 (82)	567 (82)	1155 (79)	361 (78)	981 (63)	265 (65)	389 (58)	181 (56)
White, n (%)	8190 (90)	3192 (92)	661 (96)	1312 (90)	420 (91)	1296 (83)	365 (90)	563 (85)	287 (89)
Overweight, n (%)	3509 (39)	1257 (36)	251 (36)	629 (43)	206 (45)	577 (37)	137 (34)	281 (42)	134 (41)
Current smoker, n (%)	4310 (48)	1643 (49)	358 (53)	730 (52)	232 (51)	685 (45)	175 (44)	304 (47)	153 (48)
Physical inactive, n (%)	2303 (25)	602 (17)	139 (20)	324 (22)	96 (21)	612 (39)	122 (30)	252 (38)	126 (37)
High alcohol intake, n (%)	1381 (15)	574 (17)	139 (20)	274 (19)	98 (21)	152 (10)	46 (12)	59 (9)	27 (8)
Systolic blood pressure, mmHg	123	125	123	125	125	120	119	122	120
Cholesterol, mmol/L	6.0	6.0	5.9	6.0	6.0	5.9	5.9	6.0	5.9
Nervous trouble/depression*, n (%)	617 (9)	141 (5)	61 (13)	75 (7)	68 (19)	94 (8)	41 (14)	65 (13)	59 (24)

* In a subset of the population with full information on this variable (n = 6756).

### Sleep and all-cause mortality

During the 22 years of follow-up, 804 deaths occurred (538 deaths among the 6,114 eligible men, and 266 deaths among the 2,984 eligible women). The mean age at time of death was 63 years for men and 64 years for women. Sleep duration was associated with all-cause mortality in the expected U-shaped fashion in both men and women, with higher risk of premature death associated with both short and long sleep durations (Table 2). Men who slept 6 hours versus 7 hours had a higher mortality risk (HR = 1.23; 95% CI: 1.01–1.50), but apart from this, the risk estimates were not statistically significant. Neither disturbed sleep on its own or disturbed sleep combined with short sleep were associated with all-cause mortality in men or women.

**Table 2:** All-cause mortality associated with sleep duration and disturbed sleep among 9,098 men and women free of pre-existing disease (cardiovascular disease, diabetes, cancer, respiratory disorders) from the Whitehall II study.

	Men			
	No of deaths	Age-adjusted HR (95% CI)	Multiple-adjusted† HR (95% CI)	Additional deaths† per 100,000 PY (95% CI)
Sleep length				
≤5 hours	25	1.33 (0.88; 1.99)	1.11 (0.73; 1.68)	48 (−134; 229)
6 hours	171	1.29 (1.06;1.56)	1.23 (1.01; 1.50)	69 (−4; 143)
7 hours	250	1 (Reference)	1 (Reference)	0 (Reference)
8 hours	87	1.21 (0.94; 1.54)	1.18 (0.92; 1.50)	51 (−30; 132)
>9 hours	5	1.69 (0.70; 4.09)	1.44 (0.59; 3.50)	124 (−284; 532)
**Restless, disturbed nights**				
Not at all	200	1 (Reference)	1 (Reference)	0 (Reference)
No more than usual	246	0.94 (0.78; 1.13)	0.95 (0.79; 1.14)	−17 (−82; 48)
More than usual	92	0.90 (0.70; 1.15)	0.92 (0.72; 1.17)	−24 (−99; 50)
**Joint effect**				
7–8 h sleep and no sleep disturbances	291	1 (Reference)	1 (Reference)	0 (Reference)
Disturbed sleep only	46	0.84 (0.61; 1.15)	0.85 (0.62; 1.16)	−38 (−117; 41)
≤6 h sleep only	150	1.19 (0.98; 1.45)	1.13 (0.93; 1.38)	44 (−33; 122)
≤6 h sleep & disturbed sleep	46	1.22 (0.90; 1.67)	1.18 (0.86; 1.61)	61 (−54; 175)
**Interaction**				
P-value for interaction		0.39	0.39	0.47
Additional deaths due to interaction				54 (−93; 201)

	Women			
	No of deaths	Age-adjusted HR (95% CI)	Multiple-adjusted† HR (95% CI)	Additional deaths† per 100,000 PY (95% CI)
**Sleep length**				
≤5 hours	22	1.22 (0.77; 1.92)	1.21 (0.76; 1.91)	77 (−135; 289)
6 hours	83	1.15 (0.87; 1.52)	1.14 (0.86; 1.52)	47 (−51; 146)
7 hours	151	1 (reference)	1 (reference)	0 (Reference)
8 hours	41	0.91 (0.64; 1.30)	0.91 (0.63; 1.30)	−26 (−123; 70)
>9 hours	5	1.52 (0.62; 3.73)	1.48 (0.60; 3.65)	148 (−248; 544)
**Restless, disturbed nights**				
Not at all	82	1 (Reference)	1 (Reference)	0 (Reference)
No more than usual	122	1.02 (0.77; 1.35)	1.03 (0.77; 1.36)	8 (−84; 101)
More than usual	62	0.96 (0.67; 1.33)	0.97 (0.70; 1.35)	−10 (−113; 93)
**Joint effect**				
7–8 h sleep and no sleep disturbances	124	1 (Reference)	1 (Reference)	0 (Reference)
Disturbed sleep only	32	1.02 (0.69; 1.50)	1.03 (0.70; 1.52)	10 (−98; 118)
≤6 h sleep only	76	1.27 (0.95; 1.69)	1.27 (0.95; 1.69)	88 (−22; 198)
≤6 h sleep & disturbed sleep	29	1.03 (0.68; 1.54)	1.03 (0.69; 1.55)	6 (−130; 102)
**Interaction**				
P-value for interaction		0.44	0.42	0.35
Additional deaths due to interaction				−91 (−285; 102)

† Adjusted for age, employment grade, ethnicity, and marital status.

### Sleep and cardiovascular disease mortality

During follow-up, 167 men and 54 women died due to CVD. Men who reported 9 hours or more of sleep appeared to be at higher risk of CVD mortality, but the risk estimates were based on only two CVD deaths (Table 3). Apart from this, neither short sleep nor disturbed sleep was associated with CVD mortality in men. However, men who reported *both* short and disturbed sleep were at higher risk of CVD mortality in the age-adjusted analysis (HR = 1.66; 1.01–2.73). This risk estimate was attenuated slightly after adjustment for other confounders (1.57; 0.96–2.58). Although not statistically significant, there was some indication of multiplicative (P = 0.13) and additive (*P* = 0.14) interaction between short sleep and disturbed sleep in men, with an estimated 67 additional deaths per 100,000 person years (95% CI: −23; 157) due to this interaction.

**Table 3:** (Men): Cause-specific mortality associated with sleep duration and disturbed sleep among 6,114 men free of pre-existing disease (cardiovascular disease, diabetes, cancer, respiratory disorders) from the Whitehall II study.

	CVD mortality				Cancer mortality				Other deaths			
	N*	Age-adjusted HR (95% CI)	Multiple-adjusted† HR (95% CI)	Additional deaths† per 100,000 PY (95% CI)	N*	Age-adjusted HR (95% CI)	Multiple-adjusted† HR (95% CI)	Additional deaths† per 100,000 PY (95% CI)	N*	Age-adjusted HR (95% CI)	Multiple-adjusted† HR (95% CI)	Additional deaths* per 100,000 PY (95% CI)
**Sleep length**												
≤6 hours	63	1.31 (0.96; 1.79)	1.18 (0.87; 1.63)	19 (−18; 55)	78	1.14 (0.87; 1.50)	1.12 (0.85; 1.48)	17 (−25; 59)	54	1.31 (0.94; 1.84)	1.23 (0.88; 1.74)	20 (−14; 55)
7–8 hours	102	1 (Reference)	1 (Reference)	0 (Reference)	144	1 (Reference)	1 (Reference)	0 (Reference)	88	1 (Reference)	1 (Reference)	0 (Reference)
>9 hours	2	2.19 (0.54; 8.88)	1.61 (0.40; 6.59)	62 (−195; 319)	2	1.49 (0.37; 6.02)	1.47 (0.36; 5.94)	55 (−207; 318)	1	1.23 (0.17; 8.81)	1.03 (0.14; 7.40)	−3 (−182; 177)
**Restless, disturbed nights**												
Not at all	62	1 (Reference)	1 (Reference)	0 (Reference)	87	1 (Reference)	1 (Reference)	0 (Reference)	50	1 (Reference)	1 (Reference)	0 (Reference)
No more than usual	73	0.90 (0.64; 1.26)	0.93 (0.66; 1.30)	−7 (−42; 29)	102	0.89 (0.67; 1.18)	0.89 (0.67; 1.18)	−18 (−59; 24)	68	1.04 (0.72; 1.50)	1.05 (0.73; 1.52)	5 (−29; 39)
More than usual	32	1.02 (0.66; 1.56)	1.08 (0.70; 1.66)	8 (−36; 52)	35	0.78 (0.53; 1.16)	0.77 (0.52; 1.15)	−32 (−79; 16)	25	0.98 (0.60; 1.58)	1.00 (0.61; 1.61)	1 (−36; 37)
**Joint effect**												
7–8 h sleep and no sleep disturbances	89	1 (Reference)	1 (Reference)	0 (Reference)	121	1 (Reference)	1 (Reference)	0 (Reference)	78	1 (Reference)	1 (Reference)	0 (Reference)
Disturbed sleep only	13	0.78 (0.44; 1.40)	0.82 (0.46; 1.47)	−13 (−56; 29)	23	1.01 (0.64; 1.57)	1.00 (0.64; 1.56)	1 (−54; 56)	10	0.68 (0.35; 1.31)	0.68 (0.35; 1.32)	−23 (−59; 13)
≤6 h sleep only	44	1.15 (0.80; 1.65)	1.04 (0.72; 1.49)	3 (−38; 45)	66	1.25 (0.93; 1.69)	1.23 (0.93; 1.69)	33 (−18; 83)	39	1.17 (0.80; 1.72)	1.10 (0.75; 1.62)	9 (−32; 50)
≤6 h sleep & disturbed sleep	19	1.66 (1.01; 2.73)	1.57 (0.96; 2.58)	57 (−16; 131)	12	0.76 (0.42; 1.38)	0.75 (0.41; 1.35)	−32 (−94; 29)	15	1.49 (0.86; 2.59)	1.43 (0.82; 2.48)	39 (−26; 104)
**Interaction**												
P-value for interaction		0.13	0.13	0.14		0.20	0.20	0.15		0.16	0.16	0.20
Additional deaths due to interaction				67 (−23; 157)				−66 (−155; 24)				53 (−28; 134)

* No. of cause-specific deaths.

† Adjusted for age, employment grade, ethnicity, and marital status.

Compared to women sleeping 7–8 hours per night, women who slept 6 or less hours per night showed higher risk of CVD mortality (HR = 1.81; 95% CI: 1.05; 3.10), equivalent to an additional 43 deaths per 100,000 person years (Table 4). Disturbed sleep was also associated with markedly higher risk of CVD mortality in women (HR = 3.04; 1.42–6.51), accounting for 70 additional CVD deaths per 100,000 person years (95% CI: 19; 121). Compared to women who slept 7–8 hours with no sleep disturbances, women who experienced both short and disturbed sleep had a considerably higher risk of CVD mortality (HR = 3.19; 1.52–6.72) equivalent to an additional 93 deaths per 100,000 person years. However, we found no evidence of synergy, as this effect was no more than the joint effect of the two individual risk factors (test of additive interaction, *P* = 0.88).

**Table 4:** (Women): Cause-specific mortality associated with sleep duration and disturbed sleep among 2,984 women free of pre-existing disease (cardiovascular disease, diabetes, cancer, respiratory disorders) from the Whitehall II study.

	CVD mortality				Cancer mortality				Other deaths			
	N*	Age-adjusted HR (95% CI)	Multiple-adjusted† HR (95% CI)	Additional deaths† per 100,000 PY (95% CI)	N*	Age-adjusted HR (95% CI)	Multiple-adjusted† HR (95% CI)	Additional deaths† per 100,000 PY (95% CI)	N*	Age-adjusted HR (95% CI)	Multiple-adjusted† HR (95% CI)	Additional deaths* per 100,000 PY (95% CI)
**Sleep length**												
≤6 hours	28	1.87 (1.09; 3.19)	1.81 (1.05; 3.10)	46 (2; 89)	56	1.10 (0.79; 1.53)	1.09 (0.78; 1.52)	18 (−48; 83)	18	0.81 (0.46; 1.42)	0.84 (0.48; 1.47)	−14 (−52; 25)
7–8 hours	26	1 (Reference)	1 (Reference)	0 (Reference)	91	1 (Reference)	1 (Reference)	0 (Reference)	39	1 (Reference)	1 (Reference)	0 (Reference)
>9 hours	0	n/a	n/a	n/a	3	1.64 (0.52; 5.19)	1.70 (0.53; 5.38)	118 (−201; 437)	2	1.18 (0.16; 8.63)	1.15 (0.16; 8.40)	14 (−170; 197)
**Restless, disturbed nights**												
Not at all	10	1 (Reference)	1 (Reference)	0 (Reference)	52	1 (Reference)	1 (Reference)	0 (Reference)	17	1 (Reference)	1 (Reference)	0 (Reference)
No more than usual	23	1.56 (0.74; 3.28)	1.69 (0.80; 3.56)	25 (−11; 60)	67	0.89 (0.62; 1.28)	0.88 (0.61; 1.26)	−25 (−95; 45)	31	1.24 (0.68; 2.24)	1.23 (0.68; 2.23)	17 (−28; 61)
More than usual	21	2.66 (1.25; 5.65)	3.04 (1.42; 6.51)	70 (19; 121)	31	0.76 (0.49; 1.18)	0.74 (0.47; 1.16)	−53 (−129; 24)	10	0.74 (0.34; 1.61)	0.73 (0.33; 1.61)	−17 (−61; 27)
**Joint effect**												
7–8 h sleep and no sleep disturbances	17	1 (Reference)	1 (Reference)	0 (Reference)	74	1 (Reference)	1 (Reference)	0 (Reference)	33	1 (Reference)	1 (Reference)	0 (Reference)
Disturbed sleep only	9	2.09 (0.93; 4.70)	2.36 (1.05; 5.33)	48 (−7; 103)	17	0.91 (0.53; 1.53)	0.90 (0.53; 1.53)	−17 (−96; 63)	6	0.71 (0.30; 1.70)	0.70 (0.29; 1.68)	−22 (−70; 27)
≤6 h sleep only	16	1.91 (0.96; 3.78)	1.85 (0.93; 3.68)	37 (−10; 85)	43	1.21 (0.83; 1.77)	1.21 (0.83; 1.76)	43 (−41; 127)	14	0.87 (0.45; 1.63)	0.91 (0.48; 1.70)	−8 (−57; 41)
≤6 h sleep & disturbed sleep	12	3.09 (1.48; 6.48)	3.19 (1.52; 6.72)	93 (10; 176)	13	0.77 (0.43; 1.39)	0.76 (0.42; 1.37)	−49 (−142; 45)	4	0.53 (0.19; 1.50)	0.55 (0.19; 1.55)	−38 (−92; 16)
**Interaction**												
P-value for interaction		0.65	0.58	0.88		0.40	0.39	0.30		0.83	0.84	0.84
Additional deaths due to interaction				8 (−97; 113)				−75 (−219; 68)				−8 (−88; 72)

* No. of cause-specific deaths.

† Adjusted for age, employment grade, ethnicity, and marital status.

We conducted several sensitivity analyses to assess the robustness of our findings (Table 5). We had already excluded everyone with pre-existing disease at baseline to prevent reverse causality (existing ill-health giving rise to sleep problems). Further to address this issue, we also excluded deaths occurring within the first four years of follow-up. Although this reduced the number of deaths, we found very similar results to the main analysis (Table 5). Adjustment for nervous trouble and persistent depression in a subset of the population with information on this variable (n = 6756) slightly attenuated the higher risk of CVD mortality associated with both short and disturbed sleep in women (HR = 2.35; 95% CI: 1.04–6.12), but not in men (HR = 1.87; 1.08–3.25). Further adjustments for health behaviors and CVD risk factors did not markedly change the risk estimates (Table 5).

**Table 5:** (Sensitivity analyses): Cardiovascular disease (CVD) mortality associated with the joint effects of sleep duration and disturbed sleep among 9,098 men and women free of pre-existing disease (cardiovascular disease, diabetes, cancer, respiratory disorders) from the Whitehall II study in sensitivity analyses.

	Men				Women			
	7–8 h sleep and no sleep disturbances	Disturbed sleep only	≤6 h sleep only	≤6 h sleep & disturbed sleep	7–8 h sleep and no sleep disturbances	Disturbed sleep only	≤6 h sleep only	≤6 h sleep & disturbed sleep
**4-year lag**								
No. of deaths	85	13	39	18	15	9	16	12
HR† (95% CI)	1 (reference)	0.85 (0.48;1.53)	0.97 (0.66; 1.42)	1.56 (0.94; 2.60)	1 (reference)	2.69 (1.17; 6.18)	2.11 (1.04; 4.28)	3.63 (1.69; 7.79)
**Exclusion of individuals with BMI≥25 kg/m^2^**								
No. of deaths	44	9	20	4	5	3	7	5
HR† (95% CI)	1 (reference)	1.18 (0.58; 2.43)	1.07 (0.63; 1.81)	0.74 (0.26; 2.05)	1 (reference)	2.37 (0.56; 9.95)	2.86 (0.90; 9.08)	4.01 (1.15; 14.0)
**Adjustment for depression/anxiety** *								
No. of deaths	59	7	35	17	14	7	14	8
HR† (95% CI)	1 (reference)	0.68 (0.31; 1.50)	1.16 (0.76; 1.77)	1.87 (1.08; 3.25)	1 (reference)	2.15 (0.86; 5.37)	2.06 (0.98; 4.35)	2.53 (1.04; 6.12)
**Adjustment for health behaviour (smoking, alcohol, physical activity)**								
No. of deaths	85	12	42	17	16	9	16	11
HR† (95% CI)	1 (reference)	0.83 (0.46; 1.49)	1.04 (0.71; 1.51)	1.65 (1.00; 2.73)	1 (reference)	2.47 (1.09; 5.58)	1.69 (0.83; 3.40)	2.96 (1.38; 6.38)
**Adjustment for CVD risk factors (systolic blood pressure, cholesterol, BMI)**								
No. of deaths	88	13	43	18	15	9	16	12
HR† (95% CI)	1 (reference)	0.87 (0.48; 1.56)	1.01 (0.70; 1.46)	1.46 (0.88; 2.43)	1 (reference)	2.66 (1.14; 6.18)	2.05 (1.01; 4.16)	3.81 (1.77; 8.20)

* In sub-sample with information on this variable (n = 6756).

† Adjusted for age, employment grade, ethnicity, and marital status.

Further to reduce potential confounding from sleep disorders, we also restricted the sample to those of normal weight (BMI<25 kg/m^2^). This severely reduced the sample size and especially the number of CVD deaths (to 77 in men and 20 in women). In this reduced sample of normal-weight people, no higher CVD mortality risk was found among men who experienced both short and disturbed sleep (HR = 0.74; 95% CI: 0.26–2.05), while women with short and disturbed sleep still appeared to be at markedly higher risk of CVD mortality (4.01; 1.15–14.0).

### Sleep and cancer mortality or death due to other causes

During follow-up, 224 men and 150 women died of cancer and 143 men and 59 women died of other causes (including deaths due respiratory diseases and external causes). Both men (HR = 1.47; 0.36–6.02) and women (1.70; 0.53–5.38) who slept 9 or more hours appeared to be at higher risk of cancer mortality, but the confidence intervals were very broad due to the low number of cases (Tables 3 & 4). Apart from this there were no noteworthy associations between any of the sleep measures and deaths due to cancer or other causes.

## Discussion

In a large prospective population-based study of middle-aged men and women, we found sleep duration to be associated with all-cause mortality in the expected U-shaped manner. Short sleep duration or disturbed sleep were not independently associated with CVD mortality in men, but men who reported *both* short and disturbed sleep appeared to be at higher risk. Associations with CVD mortality seemed to be different for women. Both short sleep duration and disturbed sleep were independent predictors of CVD mortality in women, and women who experienced both short and disturbed sleep, compared to those who slept 7–8 hours with no sleep disturbances, had a three times higher risk of CVD mortality, equivalent to approximately one additional death per 1000 person years. This effect equals that expected from the combination of the two independent risk factors. We found no noteworthy associations between any of the sleep measures and deaths due to cancer or other causes in men or women. Interaction analyses require large sample sizes and some of the risk estimates from the interaction analyses were based on relatively few deaths and should therefore be interpreted with caution. For this reason, we were also not able to address the interaction between long sleep duration and disturbed sleep, as we had very few people in this risk category.

Recent systematic reviews of the literature show that sleep duration is associated with mortality in a U-shaped fashion, with the lowest risk found in those who report durations of around 7 hours [2], [3]. In the Whitehall II study, both short and long sleep as well as changes in sleep duration toward either the short or long end of the sleep duration spectrum have previously been shown to be associated with higher mortality [17]. Our findings of a higher risk of all-cause mortality in those who report short or long sleep are in agreement with these previous findings.

Studies that have examined the associations between sleep and cause-specific mortality have provided mixed findings [3], [4]. Our findings add to the existing literature by demonstrating sex differences in the effect of sleep parameters on the risk of CVD mortality, in addition to addressing the combined effect of short sleep duration and disturbed sleep on cause-specific mortality for the first time. Short sleep has previously been shown to be associated with CVD-related mortality in the Whitehall II study [17], but the present findings emphasize the importance of also considering sleep disturbances, either in combination with sleep duration or as an independent risk factor for CVD mortality. These findings are in agreement with the findings of two previous studies, showing a higher risk of CHD incidence among those exposed to both short sleep duration and disturbed sleep [7], [8].

In light of previous studies, the observed sex differences in the results for CVD mortality are not surprising. We find both short sleep duration and sleep disturbances to be stronger risk factors for CVD mortality in women than in men and to account for a higher proportion of CVD deaths. Some of these differences may be explained by recently observed sex differences in the associations between sleep and cardiovascular risk factors and inflammatory markers [6], [18], [19]. In a French cohort study with annual data, sleep disturbances were associated with a higher risk of developing both hypertension and diabetes with the strongest effects observed for women [6]. Sex differences in the relation between sleep duration and hypertension have also been found previously in the Whitehall II study, with short sleep being associated with prevalence and incidence of hypertension in women only [18]. Low grade inflammation is assumed to be a factor in the development of cardiovascular disease [20], and an association between sleep duration and inflammatory markers has previously been shown for women, but not men, in the present study population [19]. The hypothalamic-pituitary-adrenal axis has also been proposed as a potential mechanism by which impaired sleep might be associated with poor health [21], and it has been shown that short sleep and sleep disturbances are independently associated with an increased cortisol awakening response and a flatter diurnal slope in cortisol secretion in the Whitehall II study, although no sex differences were observed in these associations [22].

### Limitations

Inadequate or disturbed sleep may simply be markers of existing morbidity, and it is well known that people who report shorter or longer sleep often are more likely to be in poorer overall health and have been diagnosed with medical conditions, including depression [3]. Although we cannot fully rule out this explanation, we tried to accommodate such concerns by restricting all analyses to individuals without pre-existing disease. In sensitivity analyses, we also excluded deaths within the first four years of follow-up from the statistical analyses, but found very similar results to those in the main analysis. Adjustment for self-reported nervous trouble and persistent depression in a sensitivity analyses slightly attenuated the higher risk of CVD mortality associated with short and disturbed sleep observed in women, but not in men. Although attenuated after adjustment for depression, women with short and disturbed sleep still showed more than twice the risk of CVD mortality compared to women with 7–8 hours of sleep and no sleep disturbances.

Like most large population-based studies, we lacked information on sleep apnea, a major and prevalent sleep disorder. Sleep apnea often results in disturbed sleep and also constitutes a risk factor for premature death and CVD [23], [24], making confounding by sleep apnea a concern. Since sleep apnea is less common in people of normal weight, we repeated our analyses in a subset of the population with BMI<25 kg/m^2^. In this subset of the population a higher CVD mortality risk was not found among men who experienced both short and disturbed sleep, while women with both risk factors still appeared to confer a markedly higher risk of CVD mortality. The number of CVD cases was severely reduced in this analysis, which makes it difficult to separate confounding from statistical variability. Sleep apnea often goes undiagnosed for many years and it has been estimated that as many as 75% of severe cases of sleep disordered breathing remain undiagnosed [25]. Disturbed sleep may therefore in some cases be understood as a marker of underlying sleep disorders instead of an independent risk factor.

Our information on sleep relied on two single-item self-reported questions on sleep duration and disturbed sleep, which is likely to have resulted in some misclassification. Unfortunately, it is often not feasible to obtain objective measures of sleep in large population samples, and self-reported assessments of sleep have previously been shown to be a reasonably valid estimate of quantitative sleep assessed by diary, actigraph, or polysomnography data [26]–[28]. In addition, assessments of sleep durations in the primary health care setting rely on self-reported data from patients making such simple questions relevant in terms of risk prediction. More detailed questionnaire-based information on sleep patterns including naps, sleep efficiency and validated scales for assessment of sleep disturbances would have been preferable and the lack of more detailed sleep measures may have resulted in some degree of misclassification. Finally, we were not able take changes in sleep patterns over time into account and such potential misclassification may have resulted in an underestimation of the risk estimates.

This study has also several strengths. It is the first large-scale study to describe the combined effect of short sleep duration and disturbed sleep on cause-specific mortality. Longitudinal data from a well-characterized cohort enabled us to adjust for a number of well-known confounders of the sleep-mortality association, and detailed information on pre-existing disease at baseline allowed us to restrict the study population to those without such diseases. Linkage to a nationwide death registry enabled identification of virtually all deaths and allowed for nearly complete long-term follow-up. Application of a newly proposed method to address additive interaction in survival analyses allowed us to directly estimate potential deviation from additivity of effects as well as assess the absolute effects of each sleep parameter independently and jointly, providing a clinically relevant estimate of additional deaths associated with the sleep variables.

### Conclusion

Poor sleep is a common and increasing public health problem in westernized countries. We find both short sleep duration and disturbed sleep to be strong independent indicators of CVD mortality in women. In men, evidence for a higher risk of CVD mortality in relation to these sleep parameters did not reach statistical significance, but suggests a slightly elevated risk among those exposed to both short sleep duration and disturbed sleep. Disturbed sleep may be an indicator of underlying sleep apnea, and future population-based studies may benefit from assessing disturbed sleep in addition to sleep duration in order to capture health-relevant features of inadequate sleep.
